# Development and validation of a competing risk nomogram for predicting PICC-related venous thrombosis in patients with gastrointestinal malignancies

**DOI:** 10.3389/fonc.2026.1822555

**Published:** 2026-08-05

**Authors:** Ting Cui, Jingya Li, Wei Chen, Yu Hou, Hongli Lv

**Affiliations:** 1 Interventional Radiology, Jinling Hospital, Affiliated Hospital of Medical School, Nanjing University, Nanjing, Jiangsu, China; 2 Department of Gastroenterology and Hepatology, Jinling Hospital, Affiliated Hospital of Medical School, Nanjing University, Nanjing, Jiangsu, China

**Keywords:** catheter-to-vein ratio (CVR), competing risk analysis, gastrointestinal neoplasms, nomograms, peripherally inserted central catheter (PICC), venous thrombosis

## Abstract

**Background:**

Peripherally inserted central catheter-related venous thrombosis (PICC-RVT) is an important complication in patients with gastrointestinal (GI) malignancies. Established catheter-related factors may interact with patient-level hypercoagulability and inflammation, but their combined predictive contribution in patients undergoing interventional therapy remains uncertain. This study aimed to estimate the cumulative incidence of symptomatic PICC-RVT and develop an internally validated prediction model accounting for competing events.

**Methods:**

This single-center retrospective cohort included 782 patients with GI malignancies who underwent PICC placement. The primary outcome was symptomatic PICC-RVT confirmed by ultrasonography. Death and unplanned catheter removal for non-thrombotic causes were treated as competing events. Seven candidate predictor parameters were prespecified and evaluated using Fine–Gray subdistribution hazard regression, followed by Akaike information criterion-based backward reduction. Model discrimination and calibration were assessed using the C-index and calibration curves. Internal validation was performed with 1,000 bootstrap resamples, repeating the complete model-development procedure.

**Results:**

During a median potential follow-up of 72 days, 74 patients developed symptomatic PICC-RVT. The cumulative incidence was 7.8% at 1 month and 9.2% at 3 months. Four predictors were retained in the final model: CVR >0.45, non-cavoatrial junction catheter tip position, higher baseline D-dimer, and NLR ≥3.0. The apparent C-index was 0.76 (95% CI, 0.71–0.81), and the optimism-corrected C-index was 0.75. Calibration was acceptable at 1, 2, and 3 months.

**Conclusions:**

The model quantified the combined associations of established catheter-related factors and patient-level coagulation and inflammatory markers with symptomatic PICC-RVT in patients with GI malignancies undergoing interventional therapy. These findings reinforce standardized catheter sizing and tip positioning. External validation and clinical impact assessment are required before the model is considered for clinical implementation.

## 1 Introduction

Peripherally inserted central catheters (PICCs) are widely used in oncology to provide stable long-term venous access for chemotherapy, parenteral nutrition, and supportive treatment (1, 2). In patients with gastrointestinal (GI) malignancies, PICCs may also support complex and prolonged treatment pathways associated with interventional procedures, including transarterial chemoembolization (TACE) and hepatic artery infusion chemotherapy (HAIC). Despite these clinical benefits, PICC-related venous thrombosis (PICC-RVT) remains an important complication that may cause upper-extremity pain and swelling, catheter dysfunction, interruption of anticancer treatment, and, in some cases, pulmonary embolism (1, 2).

The reported incidence of PICC-RVT in patients with cancer varies considerably, ranging from approximately 2% to more than 20%, depending on patient characteristics, diagnostic strategies, catheter practices, and follow-up duration (3). Previous studies in patients with gastric cancer have also demonstrated a clinically relevant burden of thrombotic complications, although the reported risks have varied according to the study population and outcome definition (4). Several catheter-related and patient-related risk factors have already been identified. In particular, an excessive catheter-to-vein ratio and suboptimal catheter tip position are recognized modifiable factors and are addressed in contemporary infusion therapy standards. Patient-level factors, including malignancy-associated hypercoagulability and systemic inflammation, may further contribute to thrombotic risk. However, the magnitude and combined predictive contribution of these factors may differ across cancer populations and treatment settings.

Most existing PICC-RVT prediction models have been developed in heterogeneous cancer populations or in tumor-specific cohorts that differ clinically from patients with GI malignancies undergoing interventional therapy (5, 6). Consequently, their predictive performance and calibration may not be directly transportable to this population. Patients undergoing TACE, HAIC, or related interventional treatments may have advanced tumor burden, repeated invasive treatment, treatment-associated inflammatory responses, and a substantial probability of death or non-thrombotic catheter removal during follow-up. These characteristics may alter both the observed incidence of PICC-RVT and the relative contribution of individual predictors.

An additional methodological concern is that many previous studies have not explicitly accounted for competing events that preclude the observation of PICC-RVT. In patients with advanced malignancy, death and unplanned catheter removal are clinically relevant competing events rather than ordinary non-informative censoring events. Treating these outcomes as censored observations in conventional survival analyses may overestimate the cumulative incidence of thrombosis and reduce the interpretability of predicted absolute risks (7). Competing-risk methods are therefore more appropriate for estimating PICC-RVT probability in populations with substantial competing morbidity and mortality (7, 8). The relevance of this approach lies not in redefining established catheter standards or identifying previously unknown procedural risk factors, but in quantifying the joint contribution of recognized catheter-related and patient-related variables within a population-specific risk framework.

Accordingly, this study aimed to estimate the cumulative incidence of symptomatic PICC-RVT in patients with GI malignancies undergoing interventional therapy and to develop and internally validate a competing-risk prediction model integrating prespecified catheter-related and patient-related variables. The model was intended to quantify individual risk within this specific clinical population and to provide a basis for subsequent external validation and evaluation of its incremental clinical value beyond established catheter-management standards.

## 2 Methods

### 2.1 Study design

This single-center, retrospective cohort study was conducted at the Department of Interventional Radiology, Jinling Hospital, Affiliated Hospital of Medical School, Nanjing University, to investigate the cumulative incidence and risk factors of PICC-RVT in patients with gastrointestinal malignancies. The study population comprised eligible patients who underwent interventional therapy between February 1, 2023, and December 31, 2025. The study protocol was reviewed and approved by the Institutional Review Board (IRB) of Jinling Hospital, Affiliated Hospital of Medical School, Nanjing University (Approval No.2024DZKY-081-02). Due to the retrospective nature of the analysis and the use of de-identified data, the requirement for written informed consent was waived by the IRB.

### 2.2 Study population

Potential participants were retrospectively identified through a systematic query of the hospital’s Hospital Information System (HIS) and Nursing Information System (NIS). The initial screening utilized International Classification of Diseases, Tenth Revision (ICD-10) codes for malignant neoplasms of digestive organs combined with procedural codes for interventional radiology services. To minimize selection bias, a two-step verification process was implemented: first, a preliminary dataset was generated by the Clinical Big Data Center; second, two independent researchers manually reviewed electronic medical records to verify eligibility. Agreement between reviewers was assessed, and any discordance regarding patient eligibility was adjudicated by a senior interventional radiologist and a vascular nurse specialist until consensus was reached.

Inclusion criteria consisted of the following: (1) Pathologically or cytologically confirmed diagnosis of gastric, colorectal, hepatocellular, or other gastrointestinal malignancies, uniformly staged according to the AJCC Cancer Staging Manual, 8th Edition (9) to ensure consistency across the study period; (2) Receipt of at least one session of interventional therapy (including TACE, HAIC, or portal vein stenting) during the study period; (3) Successful placement of a PICC under ultrasound guidance using the modified Seldinger technique (MST) for chemotherapy or nutritional support, adhering to the Infusion Therapy Standards of Practice (10); and (4) Availability of baseline demographic and catheter-related data.

Exclusion criteria encompassed any of the following: (1) Ultrasound-confirmed venous thrombosis in the ipsilateral upper extremity prior to catheter insertion; (2) Severe coagulation disorders (e.g., platelet count <50 × 10^9^/L) or current use of prophylactic or therapeutic doses of anticoagulants for any indication (e.g., atrial fibrillation, recent major surgery) (11); (3) Catheter dwell time less than 48 hours for reasons other than death or thrombosis (e.g., accidental dislodgment or immediate malposition), as this precludes adequate observation for catheter-related complications; (4) Incomplete follow-up records preventing the adjudication of the primary endpoint or competing events.

### 2.3 Data collection and outcome definition

Data extraction was executed using a standardized, structured logic to ensure the integrity and reliability of the predictors. Demographic and baseline clinical characteristics were retrieved from the EMR system, including age, sex, body mass index (BMI), smoking history, and the Eastern Cooperative Oncology Group (ECOG) performance status (12). Disease-specific variables comprised the primary tumor site, TNM staging, presence of distant metastasis, and the specific interventional protocol (TACE vs. HAIC/Stenting). To comprehensively adjust for hypercoagulable states, we extracted data on comorbidities (hypertension, diabetes), history of prior venous thromboembolism (VTE), and the specific administration of vesicant chemotherapy agents, particularly platinum-based compounds (cisplatin, oxaliplatin) and fluoropyrimidines (5-fluorouracil), given their varying thrombogenic potentials. Crucially, catheter-related parameters, recognized as the primary modifiable risk factors, were meticulously recorded from the nursing procedural logs. These included the catheterized vein (basilic vs. cephalic/median cubital), catheter size (French), number of puncture attempts, and the catheter tip position. The tip location was confirmed by post-procedural chest radiography. Following institutional protocol, the radiographs were formally interpreted by an attending radiologist who was blinded to the patients’ clinical data and outcomes. The optimal position was defined as the cavoatrial junction (CAJ), radiographically identified near the lower border of the right main bronchus or approximately two vertebral units below the carina, in strict accordance with the Infusion Therapy Standards of Practice (8th Edition) (2). The catheter-to-vein ratio (CVR) was calculated as the outer diameter of the catheter divided by the vein diameter. To ensure measurement standardization, pre-procedural vein diameter was measured using a high-frequency (10–14 MHz) linear array ultrasound transducer. Patients were positioned supine with the targeted arm abducted at 90°. Measurements were taken at the intended puncture site in the transverse plane, measuring the true maximum lumen from inner-wall to inner-wall (intima-intima) with a light tourniquet applied 5–10 cm proximally to induce physiological dilation (13). A CVR >0.45 was traditionally considered high risk (14). Baseline laboratory indices were defined as the most recent value obtained within 7 days prior to catheterization to reflect the immediate preoperative status (median interval between blood collection and catheter insertion: 2 days, interquartile range [IQR]: 1–3 days). These included platelet count, albumin, C-reactive protein (CRP), and D-dimer (quantified via latex-enhanced immunoturbidimetric assay, reference range <0.5 mg/L FEU). To enhance the predictive value of the model, systemic inflammatory markers were derived, specifically the neutrophil-to-lymphocyte ratio (NLR) and platelet-to-lymphocyte ratio (PLR).

The primary endpoint of this study was the incidence of symptomatic PICC-RVT, objectively confirmed by color Doppler flow imaging (CDFI). In accordance with current oncology clinical guidelines (11), routine ultrasound screening for asymptomatic thrombosis was not performed; therefore, all imaging evaluations in this cohort were strictly symptom-driven (e.g., triggered by upper extremity swelling, pain, or mechanical catheter dysfunction). Diagnostic criteria for PICC-RVT followed the standard vascular ultrasound guidelines, defined as the presence of an intraluminal echo-dense mass, incompressibility of the vein, or absence of spectral flow signals (15). The observation period was calculated from the date of catheter insertion to the occurrence of the primary event. A distinguishing feature of this study design is the rigorous classification of competing risk events. All-cause mortality and unplanned catheter removal due to non-thrombotic complications (e.g., severe catheter-related bloodstream infection, mechanical breakage, or accidental dislodgment) were defined as competing events, as their occurrence prevents the observation of the primary thrombotic endpoint. Patients who completed therapy with planned catheter removal or remained event-free at the end of the study period were considered censored observations. Follow-up duration was calculated from PICC insertion to PICC-RVT, a competing event, planned catheter removal, the last documented clinical assessment, or the end of the 90-day observation period, whichever occurred first. The median potential follow-up time for the entire cohort was estimated using the reverse Kaplan–Meier method, in which censored observations were treated as events and PICC-RVT and competing events were treated as censored.

### 2.4 Data quality control

To ensure the accuracy and reproducibility of the retrospective data, a rigorous, multi-tiered data verification protocol was implemented. Following this, a random sample of 15% of the patient records underwent independent manual abstraction by two trained vascular access nurses who were blinded to the study hypothesis. This process focused on verifying critical predictor variables (e.g., catheter tip position, CVR) and the adjudication of the primary endpoint. Inter-rater reliability was formally assessed, yielding a Cohen’s Kappa coefficient of 0.88 for categorical variables (e.g., presence of thrombosis) and an Intraclass Correlation Coefficient (ICC) of 0.92 for continuous variables. Specifically, for the critical measurement of vein diameter, the inter-rater reliability, assessed by the ICC, between two independent trained operators, assessed in a random subset of 50 patients, was 0.92 (95% CI: 0.88–0.95). Furthermore, the intra-rater ICC was evaluated to be 0.95 (95% CI: 0.91–0.97), indicating excellent measurement reproducibility in accordance with standard protocols. Any discrepancies identified were resolved by a senior interventional radiologist acting as the final adjudicator.

Missing data were assessed for each variable before model development. Among the 782 included patients, 703 (89.9%) had complete data for all variables used in the primary analysis, whereas 79 (10.1%) had at least one missing value. Variable-specific missingness ranged from 0% to 8.4%, with the highest proportions observed for baseline D-dimer (66/782, 8.4%), albumin (41/782, 5.2%), CVR (29/782, 3.7%), and CRP (24/782, 3.1%). The number and proportion of missing values for all study variables are presented in **Supplementary Table 1**. Multiple imputation by chained equations was performed under the missing-at-random assumption to avoid loss of information and precision. Continuous variables were imputed using predictive mean matching, and categorical variables were imputed using appropriate logistic or multinomial regression models. The imputation model included all candidate predictors, the primary outcome indicator, the competing-event indicator, and the Nelson–Aalen estimator of the cumulative hazard. Five imputed datasets were generated using 50 iterations per dataset. Multivariable Fine–Gray analyses were performed separately in each imputed dataset, and estimates were combined using Rubin’s rules. The imputation model included all candidate predictors and the Nelson-Aalen estimator of the cumulative hazard to ensure compatibility with the survival outcome. We generated 5 imputed datasets running 50 iterations each to account for the uncertainty associated with the missing values. All subsequent inferential analyses, including the univariable and multivariable Fine-Gray competing risk models, were performed on each of the imputed datasets. The final hazard ratios and standard errors were pooled according to Rubin’s rules to generate robust estimates that accurately reflect the underlying clinical reality.

### 2.5 Statistical analysis

Statistical analyses were performed using R software version 4.5.1 (R Foundation for Statistical Computing, Vienna, Austria). Continuous variables were expressed as means ± standard deviations (SD) for normally distributed data or medians with interquartile ranges (IQR) for skewed data, while categorical variables were presented as frequencies and percentages. Group comparisons were conducted using the Student’s t-test, Mann-Whitney U test, or Chi-square test as appropriate. Given the presence of competing events (death and non-thrombotic catheter removal), the Cumulative Incidence Function (CIF) was calculated to estimate the probability of PICC-RVT, and differences between groups were assessed using Gray’s test.

Because PICC-RVT was evaluated in the presence of competing events, the discriminatory ability of NLR and PLR was assessed using time-dependent receiver operating characteristic curves at 90 days within a competing-risk framework. Death and unplanned non-thrombotic catheter removal were treated as competing events. The optimal cutoff for each biomarker was defined as the value maximizing the Youden index, calculated as sensitivity + specificity − 1. The corresponding time-dependent area under the curve, 95% confidence interval, sensitivity, and specificity were reported. For clinical interpretability, the data-derived cutoff values were rounded to one decimal place for NLR and to the nearest integer for PLR. Seven candidate predictor parameters were prespecified before multivariable modeling according to clinical relevance, previous evidence, availability at the time of PICC placement, and avoidance of redundant information. These parameters were ECOG performance status, distant metastasis, access vein, catheter tip position, catheter-to-vein ratio, baseline D-dimer, and NLR. Univariable analyses were conducted for descriptive purposes and were not used to determine entry into the multivariable model. With 74 PICC-RVT events, the model contained 10.6 events per candidate predictor parameter, meeting the conventional benchmark of approximately 10 events per parameter, although this benchmark was not considered an absolute measure of sample-size adequacy. All seven parameters were initially entered into the Fine–Gray model, followed by backward reduction based on the Akaike information criterion to derive a parsimonious prediction model. Multicollinearity was assessed using variance inflation factors, with a value >5 indicating potentially important collinearity. Prior to modeling, multicollinearity among candidate predictors, particularly between inflammatory indices like NLR and PLR, was assessed using the Variance Inflation Factor (VIF). Variables with a VIF > 5 were considered collinear, and the most clinically relevant variable was retained to ensure model stability.

The performance of the competing risk prediction model was comprehensively evaluated. Discrimination was assessed using the time-dependent Concordance Index (C-index), which quantifies the model’s ability to correctly distinguish between patients who develop thrombosis and those who do not. Calibration was evaluated by plotting calibration curves at specific time points (1-month, 2-month, and 3-month), comparing the predicted probabilities against the observed cumulative incidence estimated by the Aalen-Johansen estimator to properly account for competing risks. Internal validation was performed using 1,000 bootstrap resamples. The complete modeling procedure, including backward predictor reduction, was repeated within each bootstrap sample to account for optimism introduced during model development. Model performance was evaluated in each bootstrap sample and subsequently in the original dataset, and the mean difference was used to estimate optimism in the C-index. Because coefficient shrinkage was incorporated neither into the original Fine–Gray estimation nor into the nomogram, no separate post-estimation shrinkage factor was applied; this limitation was considered when interpreting the internally validated model. Finally, to rigorously evaluate the robustness of our multivariable model, two predefined sensitivity analyses were conducted. First, to assess the impact of missing data handling, the model was refitted on the complete-case (CCA) cohort (excluding patients with any missing values). Second, to address potential temporal variability in biomarker levels, the model was restricted to a subgroup of patients whose laboratory assessments were performed strictly within 3 days prior to catheterization. The results of these sensitivity analyses are detailed in the **Supplementary Materials**. All statistical tests were two-sided, with a P-value < 0.05 considered statistically significant. Analyses were executed using the ‘cmprsk’, ‘riskRegression’, and ‘rms’ packages in R.

## 3 Results

### 3.1 Patient baseline characteristics and outcome distribution

**Figure 1:**
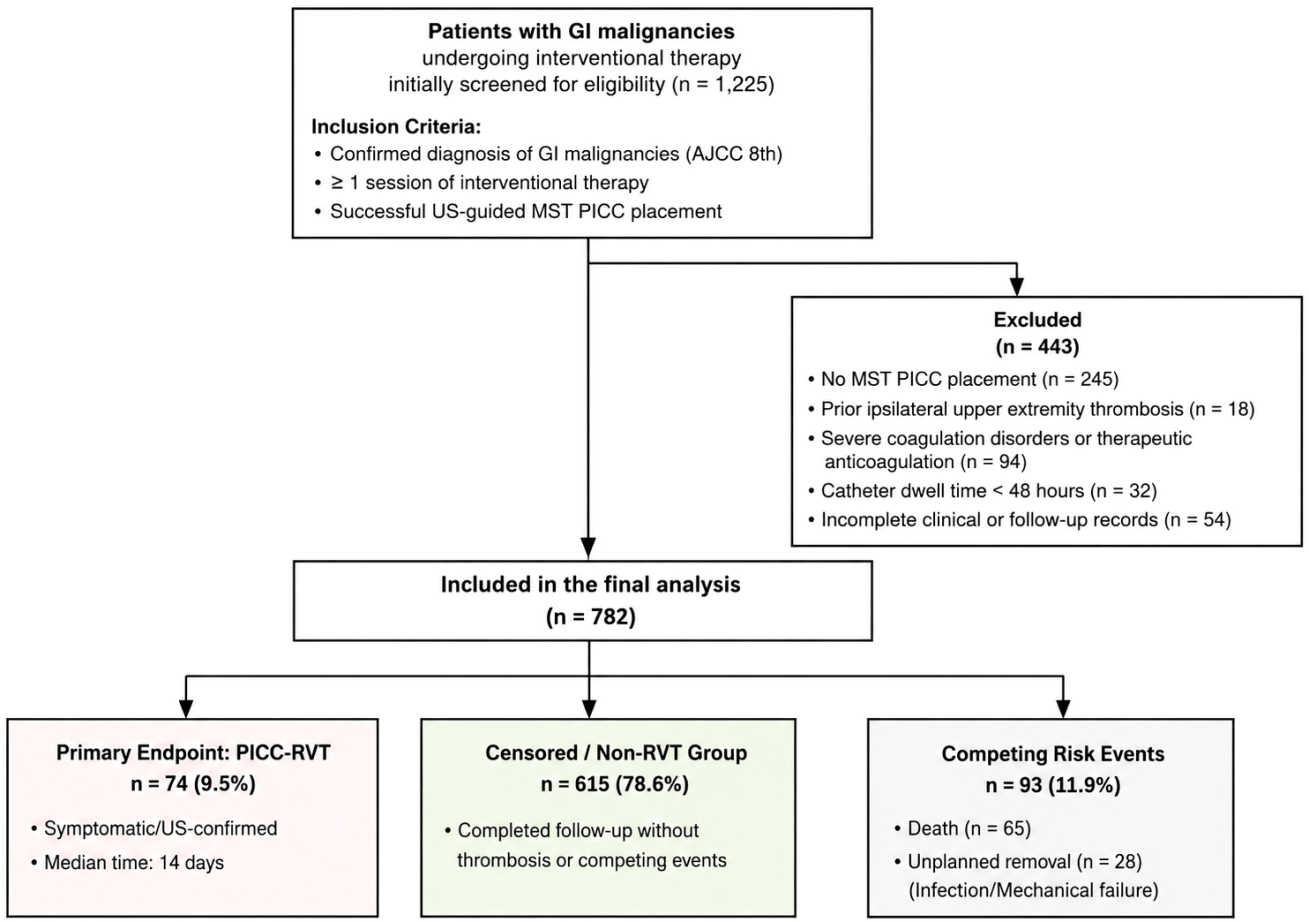
Flowchart of patient enrollment and study outcomes.

A total of 1,225 patients with gastrointestinal malignancies who underwent interventional therapy were initially screened for eligibility. According to the predefined inclusion and exclusion criteria, 443 patients were excluded: 245 did not undergo PICC placement using the modified Seldinger technique, 18 had ultrasound-confirmed venous thrombosis in the ipsilateral upper extremity before catheter insertion, 94 had severe coagulation disorders or were receiving anticoagulant therapy, 32 had a catheter dwell time of less than 48 hours, and 54 had incomplete clinical or follow-up records. Consequently, 782 patients were included in the final analytic cohort (**Figure 1**).

During follow-up, 74 patients (9.5%) developed symptomatic PICC-RVT. Competing events occurred in 93 patients (11.9%), including 65 deaths and 28 unplanned catheter removals due to severe infection or mechanical failure. The median potential follow-up time for the entire cohort, estimated using the reverse Kaplan–Meier method, was 72 days (IQR, 43–90 days). Among patients who developed PICC-RVT, the median interval from catheter insertion to thrombosis diagnosis was 14 days (IQR, 6–23 days).

Overall, 703 patients (89.9%) had complete data for all variables included in the primary analysis, whereas 79 (10.1%) had at least one missing value. Variable-specific missingness ranged from 0% to 8.4%. Baseline D-dimer had the highest proportion of missing values (8.4%), followed by albumin (5.2%), CVR (3.7%), and CRP (3.1%). Detailed variable-specific missingness is presented in **Supplementary Table 1**.

As shown in **Table 1**, age, sex, smoking history, hypertension, diabetes mellitus, and interventional protocol did not differ significantly between patients with and without PICC-RVT (all P >0.05). Patients who developed PICC-RVT had a lower BMI (P = 0.048), a higher prevalence of ECOG performance status ≥2 (P = 0.004), TNM stage IV disease (P = 0.035), and distant metastasis (P = 0.012).

**Table 1:** Baseline clinical and demographic characteristics of the study population.

Variable	Total (N = 782)	Non-RVT group (n = 708)	PICC-RVT group (n = 74)	Statistic	P-value
Demographics					
Age, years, mean ± SD	62.4 ± 10.5	62.3 ± 10.6	63.5 ± 9.8	t = -0.963	0.336
Male Sex, n (%)	485 (62.0)	438 (61.9)	47 (63.5)	χ^2^ = 0.076	0.783
BMI, kg/m^2^, mean ± SD	22.8 ± 3.1	22.9 ± 3.0	22.1 ± 3.4	t = 1.982	0.048
Smoking History, n (%)	258 (33.0)	231 (32.6)	27 (36.5)	χ^2^ = 0.448	0.503
ECOG Performance Status, n (%)				χ^2^ = 8.152	0.004
0–1	586 (74.9)	541 (76.4)	45 (60.8)		
≥ 2	196 (25.1)	167 (23.6)	29 (39.2)		
Disease Characteristics					
Primary Tumor Site, n (%)				χ^2^ = 2.145	0.342
Hepatocellular Carcinoma	352 (45.0)	320 (45.2)	32 (43.2)		
Colorectal Cancer	258 (33.0)	235 (33.2)	23 (31.1)		
Gastric Cancer	172 (22.0)	153 (21.6)	19 (25.7)		
TNM Stage, n (%)				χ^2^ = 4.452	0.035
I–III	266 (34.0)	249 (35.2)	17 (23.0)		
IV	516 (66.0)	459 (64.8)	57 (77.0)		
Distant Metastasis, n (%)	493 (63.0)	439 (62.0)	54 (73.0)	χ^2^ = 6.315	0.012
Comorbidities, n (%)					
Hypertension	211 (27.0)	193 (27.3)	18 (24.3)	χ^2^ = 0.301	0.583
Diabetes Mellitus	125 (16.0)	109 (15.4)	16 (21.6)	χ^2^ = 1.987	0.159
History of VTE, n (%)	24 (3.1)	19 (2.7)	5 (6.8)	χ^2^ = 3.654	0.056
Treatment & Medications					
Interventional Protocol, n (%)				χ^2^ = 1.452	0.228
TACE	430 (55.0)	394 (55.6)	36 (48.6)		
HAIC/Stenting	352 (45.0)	314 (44.4)	38 (51.4)		
Platinum-based agents, n (%)	469 (60.0)	421 (59.5)	48 (64.9)	χ^2^ = 0.854	0.355
Fluoropyrimidines (5-FU), n (%)	547 (70.0)	490 (69.2)	57 (77.0)	χ^2^ = 2.051	0.152
Catheter-Related Parameters					
Access Vein, n (%)				χ^2^ = 12.450	0.002
Basilic	508 (65.0)	475 (67.1)	33 (44.6)		
Cephalic/Median Cubital	274 (35.0)	233 (32.9)	41 (55.4)		
Catheter Size, n (%)				χ^2^ = 4.102	0.043
4 Fr	587 (75.1)	539 (76.1)	48 (64.9)		
5 Fr	195 (24.9)	169 (23.9)	26 (35.1)		
Puncture Attempts > 1, n (%)	188 (24.0)	165 (23.3)	23 (31.1)	χ^2^ = 2.254	0.133
Tip Position, n (%)				χ^2^ = 18.740	<0.001
Cavoatrial Junction (CAJ)	661 (84.5)	611 (86.3)	50 (67.6)		
Non-CAJ	121 (15.5)	97 (13.7)	24 (32.4)		
CVR > 0.45, n (%)	117 (15.0)	92 (13.0)	25 (33.8)	χ^2^ = 23.410	<0.001
Laboratory Indices					
Platelet Count, ×10^9^/L	185 (145–235)	184 (146–232)	192 (138–245)	Z = -0.852	0.394
Albumin, g/L	36.5 ± 4.2	36.7 ± 4.1	35.2 ± 4.5	t = 2.871	0.004
CRP, mg/L	8.5 (3.2–18.4)	8.2 (3.1–17.5)	12.4 (4.5–25.6)	Z = -3.125	0.002
D-dimer, mg/L FEU	0.68 (0.42–1.12)	0.62 (0.38–0.95)	1.85 (0.92–3.45)	Z = -7.842	<0.001
NLR	2.85 (1.95–4.12)	2.72 (1.90–3.95)	4.15 (2.85–6.24)	Z = -5.630	<0.001
PLR	145 (105–198)	140 (102–192)	185 (135–242)	Z = -4.120	<0.001

Data are presented as mean ± SD, median (interquartile range), or number (percentage). Statistical comparisons were performed using Student’s t-test, Mann-Whitney U test, or Chi-square test (χ^2^) as appropriate. Variable-specific missingness ranged from 0% to 8.4% and was handled using multiple imputation by chained equations. Abbreviations: RVT, related venous thrombosis; BMI, body mass index; ECOG, Eastern Cooperative Oncology Group; TNM, Tumor-Node-Metastasis; VTE, venous thromboembolism; TACE, transarterial chemoembolization; HAIC, hepatic arterial infusion chemotherapy; 5-FU, 5-fluorouracil; Fr, French; CAJ, cavoatrial junction; CVR, catheter-to-vein ratio; CRP, C-reactive protein; NLR, neutrophil-to-lymphocyte ratio; PLR, platelet-to-lymphocyte ratio.

In the overall cohort, 121 patients (15.5%) had a final non-CAJ catheter tip position and 117 (15.0%) had a CVR >0.45. Compared with patients without PICC-RVT, patients who developed PICC-RVT more frequently had a non-CAJ tip position (32.4% vs. 13.7%, P <0.001), a CVR >0.45 (33.8% vs. 13.0%, P <0.001), cephalic or median cubital vein access rather than basilic vein access (55.4% vs. 32.9%, P = 0.002), and a 5-Fr rather than 4-Fr catheter (35.1% vs. 23.9%, P = 0.043). Baseline albumin was lower (P = 0.004), whereas CRP, D-dimer, NLR, and PLR were higher in patients who developed PICC-RVT (all P ≤0.002).

### 3.2 Cumulative incidence of PICC-related venous thrombosis

The cumulative incidence of PICC-RVT was estimated within the competing risks framework, treating all-cause mortality and unplanned catheter removal due to non-thrombotic causes as competing events. The median potential follow-up time for the entire cohort was 72 days (IQR, 43–90 days). The risk of thrombosis increased rapidly during the early post-insertion period and subsequently tended to plateau. Specifically, the estimated cumulative incidence of PICC-RVT was 7.8% (95% CI, 6.1%–9.5%) at 30 days and increased to 9.2% (95% CI, 7.4%–11.0%) at 90 days after catheterization. Of the 74 thrombotic events, 61 (82.4%) were diagnosed within the first 28 days after PICC insertion.

**Figure 2:**
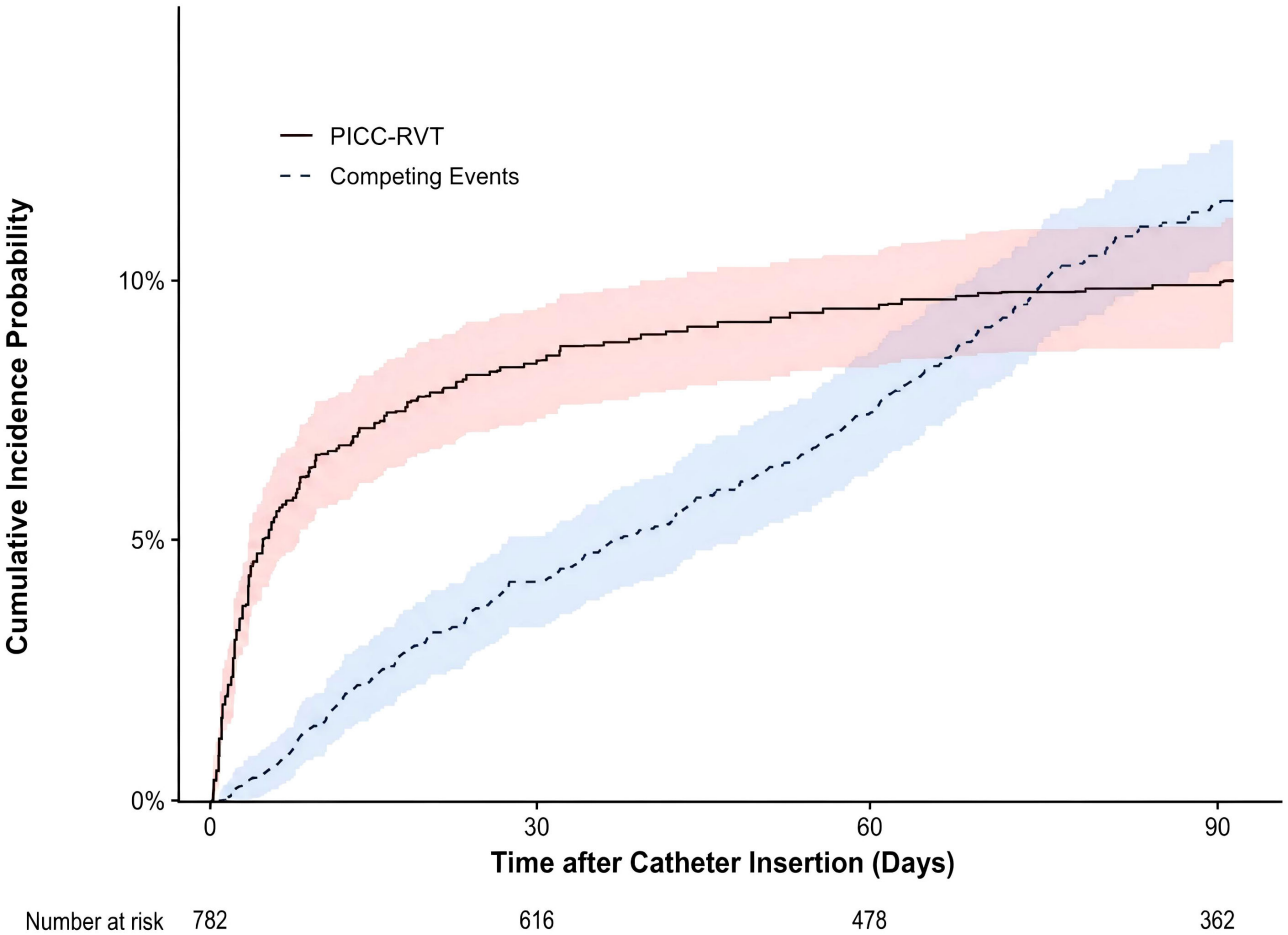
Cumulative incidence functions for symptomatic PICC-related venous thrombosis and competing events during the 90-day observation period. The solid line represents the cumulative incidence of symptomatic PICC-RVT, and the dashed line represents the cumulative incidence of competing events, defined as all-cause death or unplanned catheter removal for non-thrombotic causes. Shaded areas indicate 95% confidence intervals. The numbers at risk for the overall cohort are shown at 0, 30, 60, and 90 days. PICC-RVT, peripherally inserted central catheter-related venous thrombosis.

In contrast, the cumulative incidence of competing events demonstrated a continuous increase throughout the observation period, reaching 4.5% (95% CI: 3.2%–5.8%) at 30 days and 10.8% (95% CI: 8.9%–12.7%) at 90 days. When analyzed using the traditional Kaplan-Meier method, which treats competing events as censored data, the incidence of PICC-RVT at 90 days was overestimated at 11.5%. This discrepancy underscores the necessity of employing the competing risk framework to avoid bias in this population (**Figure 2**).

### 3.3 Univariate analysis of risk factors for PICC-RVT

The univariable Fine–Gray competing risk regression analysis was conducted to examine potential predictors associated with the cumulative incidence of PICC-related thrombosis. D-dimer was analyzed as a continuous variable, whereas NLR and PLR were categorized according to cutoff values derived from 90-day time-dependent receiver operating characteristic analyses accounting for competing events. The AUC was 0.689 (95% CI, 0.626–0.752) for NLR and 0.643 (95% CI, 0.576–0.710) for PLR. The maximum Youden index identified an optimal NLR threshold of 3.04, with a sensitivity of 67.6% and specificity of 65.1%, and an optimal PLR threshold of 149.2, with a sensitivity of 63.5% and specificity of 60.9%. For clinical presentation, these thresholds were rounded to 3.0 for NLR and 150 for PLR. Detailed results of the time-dependent ROC analyses are presented in **Supplementary Table 2**. As detailed in **Table 2**, age and sex were not significantly associated with the cumulative incidence of PICC-RVT. In contrast, an ECOG performance status ≥2 (SHR, 1.85; 95% CI, 1.12–3.05; P = 0.016) and distant metastasis (SHR, 1.92; 95% CI, 1.15–3.20; P = 0.013) were associated with a higher cumulative incidence of thrombosis. Among catheter-related variables, a non-CAJ tip position (SHR, 2.45; 95% CI, 1.55–3.88; P < 0.001) and CVR >0.45 (SHR, 2.86; 95% CI, 1.78–4.60; P < 0.001) were also associated with the outcome. Higher baseline D-dimer, NLR ≥3.0, and PLR ≥150 were significantly associated with the cumulative incidence of PICC-RVT in the univariable analyses (all P < 0.05).

**Table 2:** Univariable fine-gray competing risk analysis for PICC-related venous thrombosis.

Variable	SHR (95% CI)	P-value
Demographics		
Age (per 1-year increase)	1.01 (0.99–1.03)	0.352
Male Sex (vs. Female)	1.05 (0.65–1.68)	0.845
BMI (per 1 kg/m^2^ increase)	0.94 (0.88–1.01)	0.085
Smoking History (Yes vs. No)	1.15 (0.72–1.84)	0.562
ECOG PS (≥ 2 vs. 0–1)	1.85 (1.12–3.05)	0.016
Disease & Treatment		
Primary Tumor (Ref: Colorectal)		
Gastric Cancer	1.12 (0.65–1.92)	0.685
Hepatocellular Carcinoma	1.25 (0.78–2.01)	0.352
TNM Stage (IV vs. I–III)	1.65 (0.95–2.85)	0.075
Distant Metastasis (Yes vs. No)	1.92 (1.15–3.20)	0.013
Interventional Protocol (HAIC/Stent vs. TACE)	1.35 (0.85–2.15)	0.205
Platinum-based agents (Yes vs. No)	1.22 (0.75–1.98)	0.421
Catheter Parameters		
Access Vein (Non-Basilic vs. Basilic)	1.82 (1.15–2.88)	0.011
Catheter Size (5 Fr vs. 4 Fr)	1.55 (0.98–2.45)	0.062
Puncture Attempts (>1 vs. 1)	1.42 (0.88–2.28)	0.152
Tip Position (Non-CAJ vs. CAJ)	2.45 (1.55–3.88)	<0.001
CVR (> 0.45 vs. ≤ 0.45)	2.86 (1.78–4.60)	<0.001
Laboratory Indices		
Albumin (< 35 vs. ≥ 35 g/L)	1.62 (1.02–2.58)	0.041
CRP (> 10 vs. ≤ 10 mg/L)	1.95 (1.22–3.12)	0.005
D-dimer (per 1 mg/L FEU increase)	1.45 (1.25–1.68)	<0.001
NLR (High ≥ 3.0 vs. Low)	2.55 (1.62–4.02)	<0.001
PLR (High ≥ 150 vs. Low)	2.15 (1.35–3.42)	0.001

SHR, subdistribution hazard ratio; CI, confidence interval; ECOG PS, Eastern Cooperative Oncology Group performance status; CVR, catheter-to-vein ratio; CAJ, cavoatrial junction. The NLR and PLR cutoff values were derived from 90-day time-dependent ROC analyses accounting for competing events. The maximum Youden index identified thresholds of 3.04 for NLR and 149.2 for PLR, which were rounded to 3.0 and 150, respectively, for clinical presentation.

### 3.4 Multivariable analysis of independent predictors

Prior to multivariable modeling, multicollinearity among candidate predictors was assessed. A strong correlation was initially observed between NLR and PLR (VIF > 5). Given the robust evidence supporting NLR as a superior prognostic marker in cancer-associated thrombosis, NLR was retained while PLR was excluded. Following this exclusion, the VIFs for all remaining variables in the final model were < 2.0, indicating that multicollinearity was effectively resolved.

The initial Fine–Gray model included seven prespecified candidate predictor parameters. With 74 PICC-RVT events, this corresponded to 10.6 events per candidate parameter. After AIC-based backward reduction, four parameters were retained in the final prediction model: CVR >0.45, non-CAJ catheter tip position, baseline D-dimer, and NLR (**Table 3**). The final model therefore contained 18.5 events per retained parameter, whereas ECOG performance status, distant metastasis, and access vein were not retained in the reduced model. In the final model, CVR >0.45 was associated with a higher cumulative incidence of PICC-RVT (SHR, 2.42; 95% CI, 1.45–4.05; P < 0.001), as was a non-CAJ catheter tip position (SHR, 2.15; 95% CI, 1.32–3.50; P = 0.002). Higher baseline D-dimer was also associated with PICC-RVT (SHR per 1 mg/L FEU increase, 1.32; 95% CI, 1.12–1.55; P < 0.001), and patients with an NLR ≥3.0 had a higher subdistribution hazard than those with an NLR <3.0 (SHR, 1.95; 95% CI, 1.18–3.22; P = 0.009). The final model yielded a C-index of 0.76 (95% CI, 0.71–0.81).

**Table 3:** Reduced multivariable fine–gray competing risk model for PICC-related venous thrombosis.

Variable	Adjusted SHR (95% CI)	P-value	VIF
Catheter-to-vein ratio			1.07
≤0.45	Reference		
>0.45	2.38 (1.43–3.97)	0.001	
D-dimer, per 1 mg/L FEU increase	1.30 (1.11–1.53)	0.001	1.21
Catheter tip position			1.10
CAJ	Reference		
Non-CAJ	2.11 (1.30–3.43)	0.003	
Neutrophil-to-lymphocyte ratio			1.32
<3.0	Reference		
≥3.0	1.91 (1.16–3.15)	0.011	

Seven candidate predictor parameters were initially included in the multivariable Fine–Gray model: ECOG performance status, distant metastasis, access vein, catheter tip position, catheter-to-vein ratio, baseline D-dimer, and NLR. With 74 PICC-RVT events, this corresponded to 10.6 events per candidate parameter. Four parameters were retained after AIC-based backward reduction. The complete model-development procedure was repeated during bootstrap internal validation. SHR, subdistribution hazard ratio; CI, confidence interval; VIF, variance inflation factor; CAJ, cavoatrial junction; CVR, catheter-to-vein ratio; NLR, neutrophil-to-lymphocyte ratio; FEU, fibrinogen-equivalent units.

The robustness of these independent predictors was further confirmed by two rigorous sensitivity analyses. When refitting the Fine-Gray model on the complete-case cohort (n = 703) and on the restricted temporal cohort (laboratories drawn ≤ 3 days, n = 645), all four predictors—CVR > 0.45, non-CAJ tip position, elevated D-dimer, and high NLR—retained their statistical significance and demonstrated highly consistent subdistribution hazard ratios (all P < 0.05). Detailed results of the primary model and both sensitivity analyses are presented side-by-side in **Supplementary Table 3**.

### 3.5 Construction and validation of the prediction nomogram

**Figure 3:**
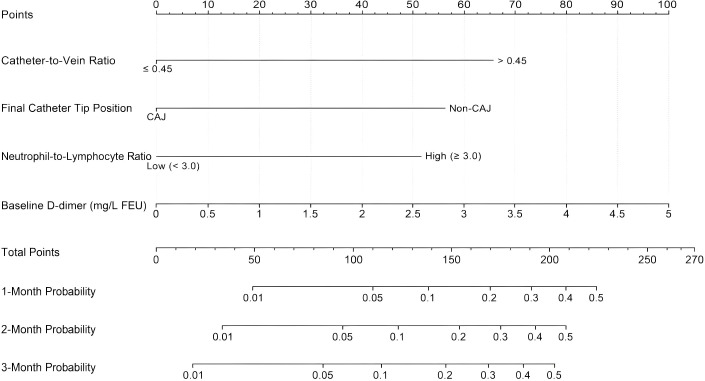
Nomogram for estimating the 1-, 2-, and 3-month cumulative incidence of symptomatic PICC-related venous thrombosis. The nomogram was derived from the final Fine–Gray competing-risk model incorporating catheter-to-vein ratio, final catheter tip position, baseline D-dimer, and neutrophil-to-lymphocyte ratio. For each predictor, the corresponding points are assigned and summed to obtain the total score, which is then mapped to the predicted cumulative incidence at 1, 2, and 3 months. CAJ, cavoatrial junction; FEU, fibrinogen-equivalent units.

To facilitate the clinical application of the predictive model, a prognostic nomogram was constructed integrating the four independent predictors identified in the multivariable Fine-Gray analysis: catheter tip position, CVR, baseline D-dimer, and NLR (**Figure 3**). In this graphical tool, each predictor is assigned a specific point value on the top scale based on its regression coefficient. The total score, calculated by summing the individual points for each patient, corresponds to the predicted cumulative incidence probabilities of PICC-RVT at 1 month, 2 months, and 3 months on the bottom scales.

**Figure 4:**
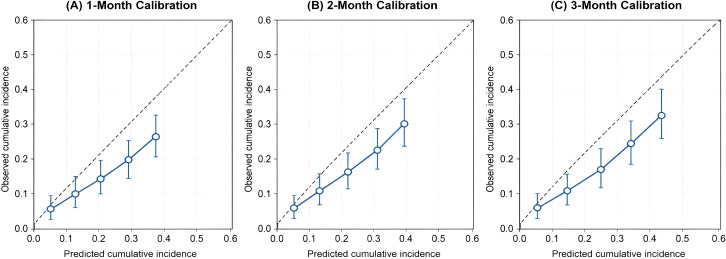
Calibration of the final competing-risk model for predicting symptomatic PICC-related venous thrombosis at 1 month **(A)**, 2 months **(B)**, and 3 months **(C)**. The x-axis represents model-predicted cumulative incidence, and the y-axis represents cumulative incidence observed using the Aalen–Johansen estimator. The dashed 45-degree line indicates perfect calibration. Circles represent grouped estimates, and vertical error bars indicate 95% confidence intervals. Calibration was evaluated using 1,000 bootstrap resamples.

The performance of the nomogram was evaluated through both discrimination and calibration metrics. The model demonstrated a C-index of 0.76 (95% CI: 0.71–0.81) on the original cohort, indicating good discriminative ability in distinguishing patients at high risk of thrombosis from those at low risk. **Figure 4** displays the calibration curves for the 1-month, 2-month, and 3-month cumulative incidence of PICC-RVT. The plots demonstrated good agreement between the nomogram-predicted probabilities and the actual observed frequencies derived from the Aalen-Johansen estimates, with the calibration lines closely approximating the ideal 45-degree diagonal reference line.

Internal validation was conducted using 1,000 bootstrap resamples, with the complete model-development procedure repeated in each resample. The apparent C-index was 0.76, and the bootstrap-estimated optimism was 0.01, resulting in an optimism-corrected C-index of 0.75. The small difference between the apparent and corrected estimates suggested limited optimism in discrimination. No separate post-estimation coefficient shrinkage factor was applied; therefore, the findings should be interpreted as internally validated performance estimates pending external validation.

### 3.5 Incremental predictive value of patient-level biomarkers

The catheter-factor model containing CVR and catheter tip position yielded a C-index of 0.68 (95% CI, 0.62–0.74). After baseline D-dimer and NLR were added, the C-index increased to 0.76 (95% CI, 0.71–0.81). The extended model also showed lower prediction error than the catheter-factor model, with Brier scores decreasing from 0.066 to 0.058 at 30 days and from 0.079 to 0.069 at 90 days. The likelihood-ratio test indicated improved model fit after inclusion of D-dimer and NLR (χ^2^ = 18.6, P <0.001). Calibration plots showed closer agreement between predicted and observed cumulative incidence for the extended model at both time points. Detailed comparisons are presented in **Supplementary Table 4**.

## 4 Discussion

In this single-center retrospective cohort of patients with gastrointestinal malignancies undergoing interventional therapy, the 90-day cumulative incidence of symptomatic PICC-RVT was 9.2%. The cumulative incidence estimated using the conventional Kaplan–Meier method was approximately 2.3 percentage points higher than that obtained within the competing-risk framework. The final model retained four predictors: CVR >0.45, non-CAJ catheter tip position, elevated baseline D-dimer, and NLR ≥3.0. The model yielded a C-index of 0.76 and showed acceptable calibration during internal validation. These findings quantify the combined contribution of established catheter-related factors and patient-level coagulation and inflammatory markers in this specific clinical population.

Accounting for competing events was important because patients with advanced gastrointestinal malignancies undergoing interventional procedures commonly have substantial disease burden, comorbidity, and mortality risk (16). In this setting, death and non-thrombotic catheter removal prevent subsequent observation of PICC-RVT and therefore should not be treated as ordinary non-informative censoring events (17). Conventional Kaplan–Meier analysis may overestimate event probability when competing events are present (18). By estimating the cumulative incidence function and applying Fine–Gray regression, the present analysis provided absolute risk estimates that more directly reflected the observed clinical course of this population. This approach is consistent with recommended methods for oncological studies in which competing outcomes are clinically relevant (19). Its contribution lies in the appropriate quantification of PICC-RVT probability in this treatment setting rather than in methodological novelty.

CVR and catheter tip position are established, modifiable determinants of catheter-related thrombosis and should not be interpreted as newly identified procedural risk factors. A catheter occupying a larger proportion of the venous lumen may reduce blood flow, increase local stasis, and promote thrombus formation, consistent with the hemodynamic component of Virchow’s triad (20, 21). Current infusion therapy standards recommend selecting the smallest catheter capable of delivering the prescribed treatment, limiting the catheter-to-vein ratio, and positioning the catheter tip in the recommended central venous location to reduce mechanical and thrombotic complications (22–24). The updated 2024 Infusion Therapy Standards of Practice further reinforce the importance of appropriate catheter sizing and optimal tip positioning (24). Similarly, catheter tips positioned proximal to the CAJ may be exposed to lower blood flow and less rapid dilution of infused agents than tips located in the recommended central position, thereby increasing endothelial irritation and thrombotic risk (25). Accurate tip assessment and correction of malposition remain important elements of PICC management (26). Accordingly, the present findings reinforce existing catheter-placement principles rather than establishing new procedural standards. Their relevance is the population-specific estimation of effect sizes and their integration with patient-level predictors in a competing-risk model.

The presence of CVR >0.45 and non-CAJ tip positions in a proportion of this cohort should also be interpreted cautiously. These observations may reflect variation in real-world practice, limited venous anatomy, catheter requirements, technical difficulties, or deviations from recommended procedures. Because the retrospective records did not systematically document why a CVR >0.45 or a proximal non-CAJ tip position was retained, the present study could not distinguish anatomical or treatment-related constraints from potentially modifiable practice variation. The model should therefore not be used to normalize or compensate for avoidable deviations from catheter-placement standards. Adherence to recommended catheter sizing, vein selection, and tip positioning remains the primary preventive strategy. Risk prediction, if subsequently validated, should be considered complementary to—not a substitute for—standardized insertion and post-placement assessment.

D-dimer and NLR provided patient-level information beyond catheter-related characteristics. Elevated D-dimer reflects increased fibrin formation and degradation and is commonly associated with malignancy-related hypercoagulability, including the thrombotic manifestations traditionally described in Trousseau syndrome (27). Tumor-derived procoagulant activity and vascular injury associated with anticancer treatments, including platinum compounds and fluoropyrimidines, may further enhance coagulation activation (28). NLR reflects the balance between systemic neutrophilic inflammation and lymphocyte-mediated immune status. Neutrophil activation may contribute to thrombosis through the release of neutrophil extracellular traps, which provide a scaffold for platelet adhesion, erythrocyte accumulation, and coagulation activation (29, 30). The observed association between elevated NLR and PICC-RVT is also consistent with previous evidence linking NLR to venous thromboembolism (31). However, these findings should be interpreted as predictive associations rather than proof of a specific causal mechanism. The incremental model comparison further indicated that adding D-dimer and NLR to CVR and tip position improved discrimination and reduced prediction error, suggesting that the model captures patient-level thrombotic susceptibility in addition to catheter-placement characteristics.

The potential clinical role of the model should remain limited at this stage. Its current value is primarily as a research-based method for estimating individual risk and examining whether established catheter-related variables and routinely available biomarkers can be combined within a competing-risk framework. The model should not yet be used to determine prophylactic anticoagulation, routine ultrasound frequency, or other management decisions, because it has not undergone external validation or clinical impact evaluation. Current oncology guidance recommends that thromboprophylaxis decisions incorporate overall venous thromboembolism risk, bleeding risk, treatment context, and patient preferences rather than rely on a single unvalidated prediction model (11). If the model demonstrates acceptable transportability in independent cohorts, it may provide supplementary information for identifying patients who remain at increased risk despite standardized catheter management. Whether model-guided surveillance or intervention improves outcomes and resource allocation requires prospective impact studies (32).

Several strengths of this study should be considered. The competing-risk analysis provided a more appropriate estimate of PICC-RVT probability in a population with clinically relevant mortality and catheter-removal risks. The study focused on a relatively homogeneous group of patients with gastrointestinal malignancies undergoing interventional treatment, thereby reducing some of the clinical heterogeneity present in broader oncology cohorts. In addition, the model combined catheter-related parameters with routinely available coagulation and inflammatory markers, and the complete model-development process was evaluated using bootstrap internal validation.

Several limitations remain. First, the retrospective design may have introduced selection bias, information bias, and incomplete ascertainment of clinical practices, despite standardized data extraction and quality-control procedures. Second, the study was conducted at a single center and lacked an external validation cohort. Internal bootstrap validation estimates optimism within the derivation data but cannot establish transportability. Differences across institutions in case mix, tumor distribution, catheter-selection practices, tip-confirmation procedures, nursing protocols, and symptom-triggered ultrasound use may affect predictor effects, baseline risk, discrimination, and calibration. The model may therefore partly capture local practice patterns and should not be implemented elsewhere without independent validation and, if necessary, recalibration. Third, the reasons for retaining a CVR >0.45 or a non-CAJ tip position were not systematically recorded, preventing distinction between anatomical or treatment-related constraints and potentially avoidable deviations from recommended practice. Fourth, only baseline D-dimer and NLR were evaluated; changes in coagulation and inflammatory status during treatment were not captured. Fifth, although 74 thrombotic events provided approximately 10.6 events per prespecified candidate predictor parameter and the complete modeling procedure was assessed using bootstrap resampling, the event count remained moderate. Residual overfitting and instability in predictor selection cannot be excluded, particularly because no separate post-estimation coefficient shrinkage was applied. Finally, ultrasound assessment was symptom-driven rather than routinely scheduled. Asymptomatic PICC-RVT may therefore have been missed, leading to underestimation of the total thrombotic burden and limiting applicability to settings that use systematic screening.

## 5 Conclusion

In patients with gastrointestinal malignancies undergoing interventional therapy, symptomatic PICC-RVT occurred predominantly during the early period after catheter insertion. The competing-risk model quantified the joint associations of two established catheter-related factors—CVR >0.45 and non-CAJ tip position—and two patient-level markers, baseline D-dimer and NLR, with the cumulative incidence of PICC-RVT. These findings reinforce the importance of standardized catheter sizing and tip positioning while indicating that patient-related hypercoagulability and inflammation may contribute additional predictive information. The model should currently be regarded as an internally validated research tool. Its incremental value, calibration, transportability, and clinical impact require confirmation in independent multicenter cohorts before it is considered for clinical implementation.

## Data Availability

The original contributions presented in the study are included in the article/**Supplementary Material**. Further inquiries can be directed to the corresponding author.
