# Supplementary material for: Development and validation of a competing risk nomogram for predicting PICC-related venous thrombosis in patients with gastrointestinal malignancies
Source: Front Oncol. 2026 Aug 5;16:1822555. doi: 10.3389/fonc.2026.1822555 (PMC13485509; doi:10.3389/fonc.2026.1822555)
Supplement: Supplementary file 1 [file Table1.docx]

**Supplementary Table S1.** Number and proportion of missing values for study variables before multiple imputation

| **Variable** | **Missing, n** | **Missing, %** |
| --- | --- | --- |
| Age | 0 | 0 |
| Sex | 0 | 0 |
| Body mass index | 4 | 0.5 |
| Smoking history | 0 | 0 |
| ECOG performance status | 3 | 0.4 |
| Primary tumor site | 0 | 0 |
| TNM stage | 0 | 0 |
| Distant metastasis | 0 | 0 |
| Hypertension | 0 | 0 |
| Diabetes mellitus | 0 | 0 |
| History of venous thromboembolism | 0 | 0 |
| Interventional protocol | 0 | 0 |
| Platinum-based agent use | 0 | 0 |
| Fluoropyrimidine use | 0 | 0 |
| Access vein | 0 | 0 |
| Catheter size | 0 | 0 |
| Number of puncture attempts | 0 | 0 |
| Catheter tip position | 0 | 0 |
| Catheter-to-vein ratio | 29 | 3.7 |
| Platelet count | 8 | 1.0 |
| Albumin | 41 | 5.2 |
| C-reactive protein | 24 | 3.1 |
| D-dimer | 66 | 8.4 |
| Neutrophil-to-lymphocyte ratio | 11 | 1.4 |
| Platelet-to-lymphocyte ratio | 11 | 1.4 |
| PICC-RVT outcome | 0 | 0 |
| Competing-event status | 0 | 0 |
| Follow-up time | 0 | 0 |

Notes: Missingness was assessed in the full analytic cohort of 782 patients before multiple imputation. A total of 703 patients (89.9%) had complete data for all variables included in the primary analysis, and 79 patients (10.1%) had at least one missing value. Because missingness overlapped across variables within individual patients, the sum of variable-specific missing counts exceeds the number of patients with any missing data. ECOG, Eastern Cooperative Oncology Group; TNM, tumor–node–metastasis; PICC-RVT, peripherally inserted central catheter-related venous thrombosis.

**Supplementary Table S2. Time-dependent ROC analysis of NLR and PLR for 90-day PICC-related venous thrombosis**

| **Biomarker** | **AUC (95% CI)** | **Optimal cutoff** | **Youden index** | **Sensitivity, %** | **Specificity, %** | **Cutoff used in analysis** |
| --- | --- | --- | --- | --- | --- | --- |
| NLR | 0.689 (0.626–0.752) | 3.04 | 0.327 | 67.6 | 65.1 | 3.0 |
| PLR | 0.643 (0.576–0.710) | 149.2 | 0.244 | 63.5 | 60.9 | 150 |

Notes: Time-dependent ROC analyses were performed at 90 days with death and unplanned non-thrombotic catheter removal treated as competing events. Optimal cutoffs were selected by maximizing the Youden index. Cutoff values were rounded for clinical interpretability before categorical Fine–Gray regression. AUC, area under the curve; CI, confidence interval; NLR, neutrophil-to-lymphocyte ratio; PLR, platelet-to-lymphocyte ratio; PICC, peripherally inserted central catheter.

**Supplementary Table S3.** Multivariable Fine-Gray Competing Risk Models: Primary and Sensitivity Analyses

| ****Predictor**** | ****Primary Model (MICE) (N = 782)**** | ****Sensitivity Model 1: Complete-Case Analysis (N = 703)**** | ****Sensitivity Model 2: Biomarker Window ≤ 3 days (N = 645)**** |
| --- | --- | --- | --- |
| CVR > 0.45 |  |  |  |
| Adjusted SHR (95% CI) | 2.42 (1.45–4.05) | 2.45 (1.42–4.18) | 2.40 (1.38–4.12) |
| P-value | < 0.001 | 0.001 | 0.002 |
| D-dimer (per 1 mg/L FEU) |  |  |  |
| Adjusted SHR (95% CI) | 1.32 (1.12–1.55) | 1.35 (1.14–1.59) | 1.34 (1.10–1.62) |
| P-value | < 0.001 | < 0.001 | 0.003 |
| Catheter Tip Position (Non-CAJ) |  |  |  |
| Adjusted SHR (95% CI) | 2.15 (1.32–3.50) | 2.18 (1.30–3.62) | 2.12 (1.25–3.55) |
| P-value | 0.002 | 0.003 | 0.005 |
| NLR (≥ 3.0) |  |  |  |
| Adjusted SHR (95% CI) | 1.95 (1.18–3.22) | 1.98 (1.15–3.35) | 1.91 (1.15–3.18) |
| P-value | 0.009 | 0.012 | 0.012 |

Notes: MICE, multiple imputation by chained equations; CCA, complete-case analysis; SHR, subdistribution hazard ratio; CI, confidence interval; CVR, catheter-to-vein ratio; CAJ, cavoatrial junction; NLR, neutrophil-to-lymphocyte ratio. In Sensitivity Model 1, patients with any missing baseline values were excluded. In Sensitivity Model 2, the cohort was restricted to patients who had laboratory tests completed within 3 days prior to catheter placement.

**Supplementary Table S4.** Incremental predictive performance of patient-level biomarkers beyond catheter-related factors

| **Performance measure** | **Catheter-factor model** | **Extended model** | **Difference or test** |
| --- | --- | --- | --- |
| Predictors | CVR, tip position | CVR, tip position, D-dimer, NLR | — |
| C-index | 0.68 (0.62–0.74) | 0.76 (0.71–0.81) | Δ = 0.08 |
| 30-day Brier score | 0.066 | 0.058 | Δ = −0.008 |
| 90-day Brier score | 0.079 | 0.069 | Δ = −0.010 |
| Likelihood-ratio test | Reference | χ² = 18.6 | P <0.001 |

Notes: The catheter-factor model included CVR and catheter tip position. The extended model additionally included baseline D-dimer and NLR. Lower Brier scores indicate lower prediction error. The likelihood-ratio test assessed whether addition of D-dimer and NLR improved overall model fit. CVR, catheter-to-vein ratio; NLR, neutrophil-to-lymphocyte ratio; CI, confidence interval.
